# Supplementary material for: Supplementation of a High-Fat Diet with Pentadecylresorcinol Increases the Representation of Akkermansia muciniphila in the Mouse Small and Large Intestines and May Protect against Complications Caused by Imbalanced Nutrition
Source: Int J Mol Sci. 2024 Jun 15;25(12):6611. doi: 10.3390/ijms25126611 (PMC11204153; doi:10.3390/ijms25126611)
Supplement: Supplementary file 1 [file ijms-25-06611-s001.zip › Supplementary Figures.pdf]

Supplementary figures

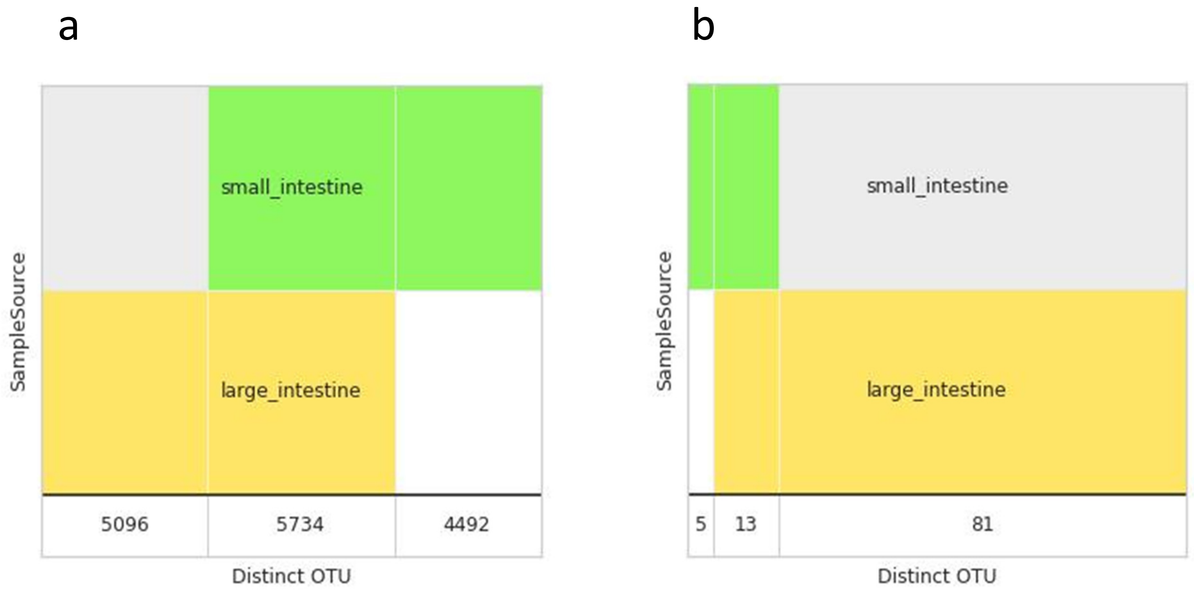

Supplementary Figure S1. Venn diagrams showing the shared and unique OTUs across SIM and LIM.

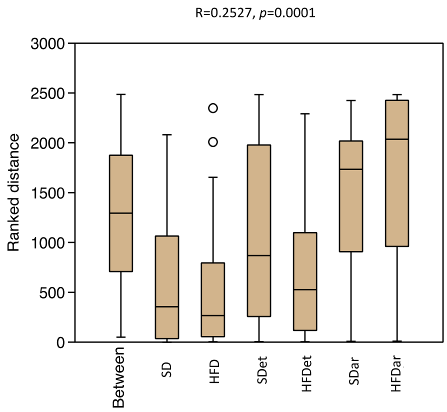

Supplementary Figure S2. ANOSIM based on Bray–Curtis distances evaluated for the SIM and LIM paired samples for the six groups according to diet type. SD, standard diet; HFD, high-fat diet; SDet, standard diet with solvent addition; HFDet, high-fat diet with solvent addition; SDar, standard diet with C15 supplementation; HFDar, high-fat diet with C15 supplementation.

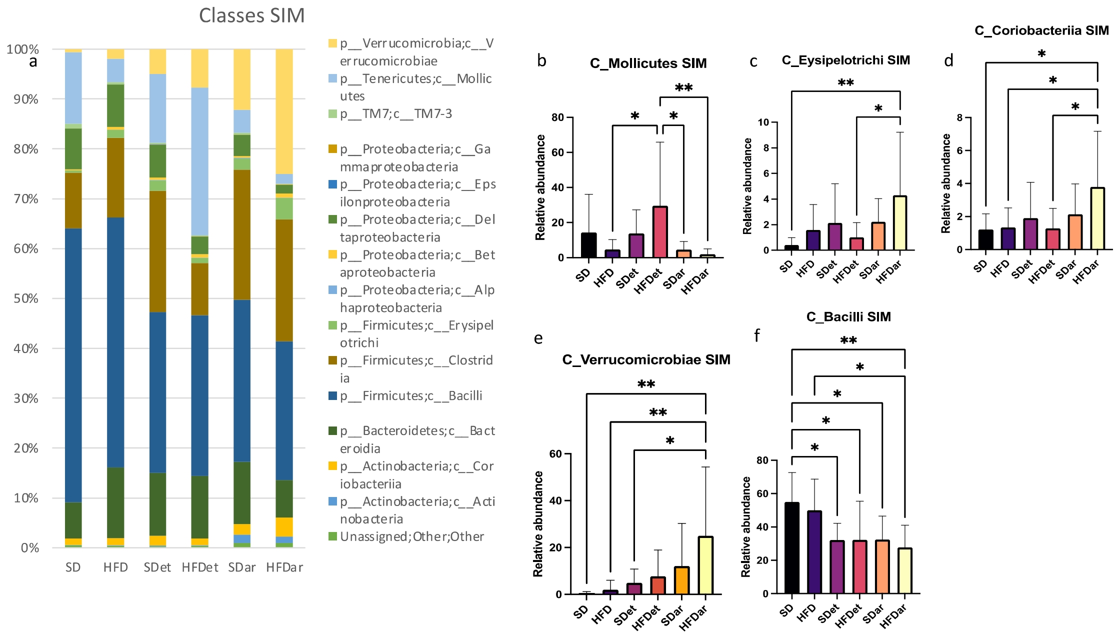

Supplementary Figure S3. Effects of different diet types on the mouse SIM community. (**a**) Class level, (**b**) *Mollicutes*, (**c**) *Eisypelotrichi*, (**d**) *Coriobacteriia,* (**e**) *Verrucomicrobiae,* and (**f**) *Bacilli.* **P* < 0.05, ***P* < 0.01. Comparisons were performed by one-way analysis of variance followed by Tukey's multiple comparison test. SD, standard diet; HFD, high-fat diet; SDet, standard diet with solvent addition; HFDet, high-fat diet with solvent addition; SDar, standard diet with C15 supplementation; HFDar, high-fat diet with C15 supplementation.

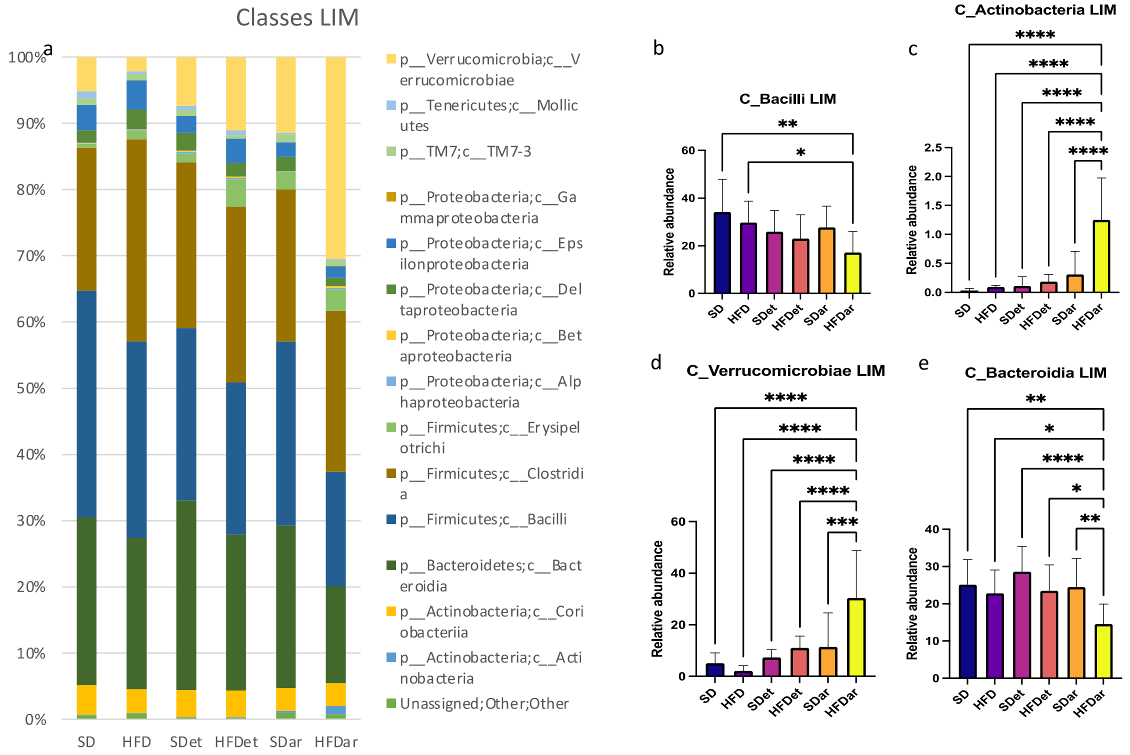

Supplementary Figure S4. Effects of different types of diet on the bacterial community of mouse LIM. (**a**) Class level; (**b**) *Bacilli*, (**c**) *Actinobacteria,* (**d**) *Verrucomicrobiae,* and (**e**) *Bacteroidia.* **P* < 0.05, ***P* < 0.01, ****P* < 0.001, *****P* < 0.0001. Comparisons were performed by one-way analysis of variance followed by Tukey's multiple comparison test. SD, standard diet; HFD, high-fat diet; SDet, standard diet with solvent addition; HFDet, high-fat diet with solvent addition; SDar, standard diet with C15 supplementation; HFDar, high-fat diet with C15 supplementation.

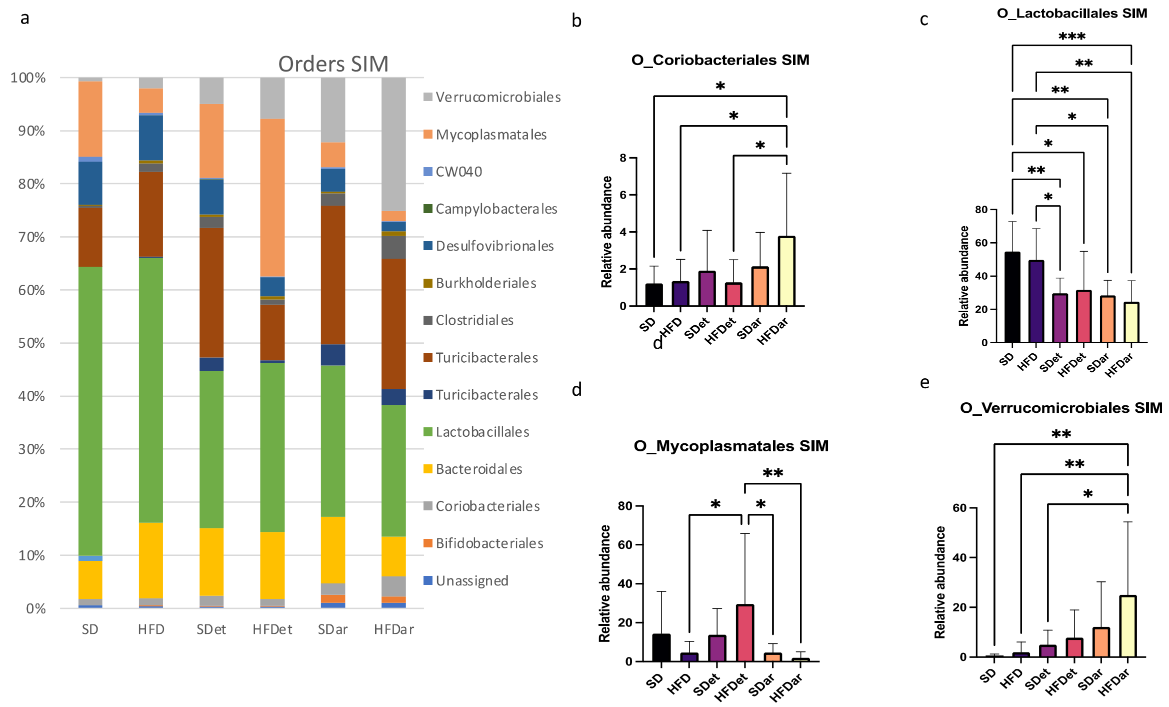

Supplementary Figure S5. Effects of different diet types on the mouse SIM community. (**a**) Order level, (**b**) *Coriobacteriales*, (**c**) *Lactobacillales,* (**d**) *Mycoplasmatales,* and (**e**) *Verrucomicrobiales.* **P* < 0.05, ***P* < 0.01, ****P* < 0.001. Comparisons were performed by one-way analysis of variance followed by Tukey's multiple comparison test. SD, standard diet; HFD, high-fat diet; SDet, standard diet with solvent addition; HFDet, high-fat diet with solvent addition; SDar, standard diet with C15 supplementation; HFDar, high-fat diet with C15 supplementation.

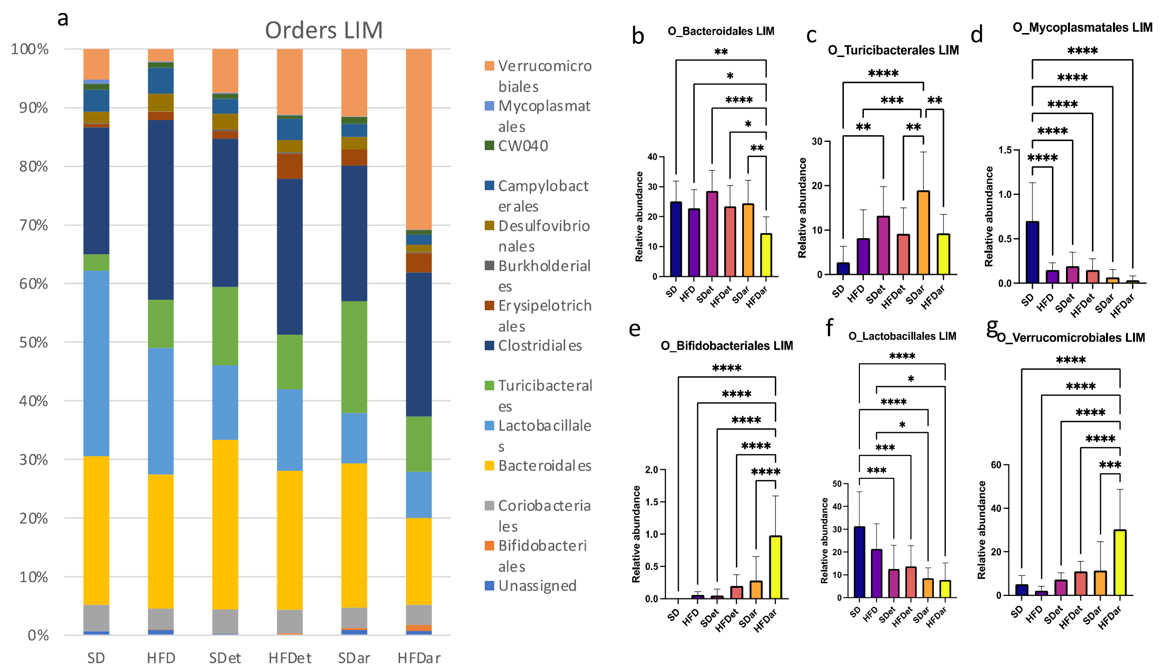

Supplementary Figure S6. Effects of different types of diet on the mouse LIM community (**a**) Order level, (**b**) *Bacteroidales*, (**c**) *Turicibacterales,* (**d**) *Mycoplasmatales,* (**e**) *Bifidobacteriales,* (**f**) *Lactobacillales,* and (**g**) *Verrucomicrobiales.* *$P < 0.05$, **$P < 0.01$, ***$P < 0.001$, ****$P < 0.0001$. Comparisons were performed by one-way analysis of variance followed by Tukey's multiple comparison test. SD, standard diet; HFD, high-fat diet; SDet, standard diet with solvent addition; HFDet, high-fat diet with solvent addition; SDar, standard diet with C15 supplementation; HFDar, high-fat diet with C15 supplementation.

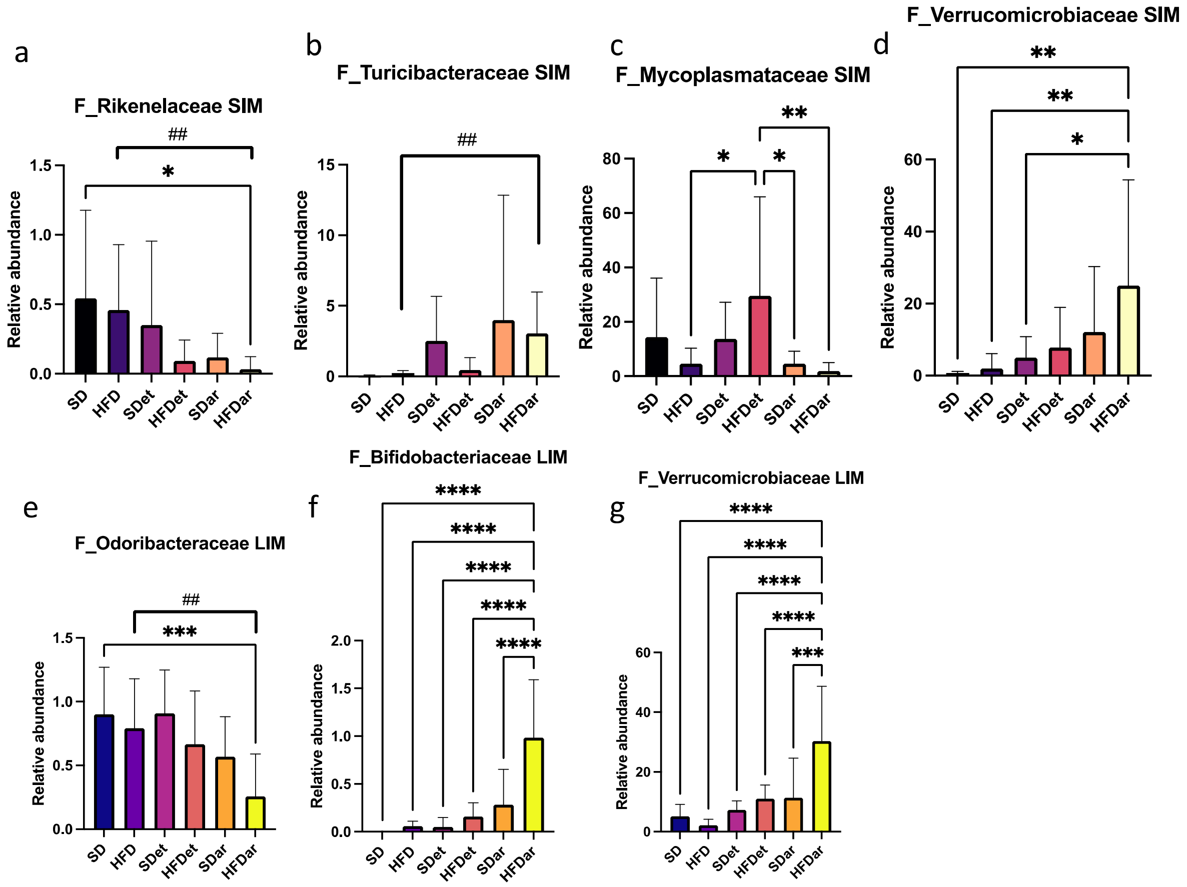

Supplementary Figure S7. Effects of different diet types on mouse SIM and LIM communities at the family level. (**a**) *Rikenelaceae* in SIM, (**b**) *Turicibacteraceae* in SIM, (**c**) *Mycoplasmataceae* in SIM, (**d**) *Verrucomicrobiaceae* in SIM*,* (**e**) *Odoribacteraceae* under LIM, (**f**) *Bifidobacteriaceae* in LIM, (**g**) *Verrucomicrobiaceae* in LIM. *$P < 0.05$, **$P < 0.01$, ***$P < 0.001$, ****$P < 0.0001$ for ANOVA; ## $P< 0.01$ for Welch's t test. Comparison was carried out using one-way analysis of variance followed by Tukey's multiple comparison test or Welch's t test. SD, standard diet; HFD, high-fat diet; SDet, standard diet with solvent addition; HFDet, high-fat diet with solvent addition; SDar, standard diet with C15 supplementation; HFDar, high-fat diet with C15 supplementation.

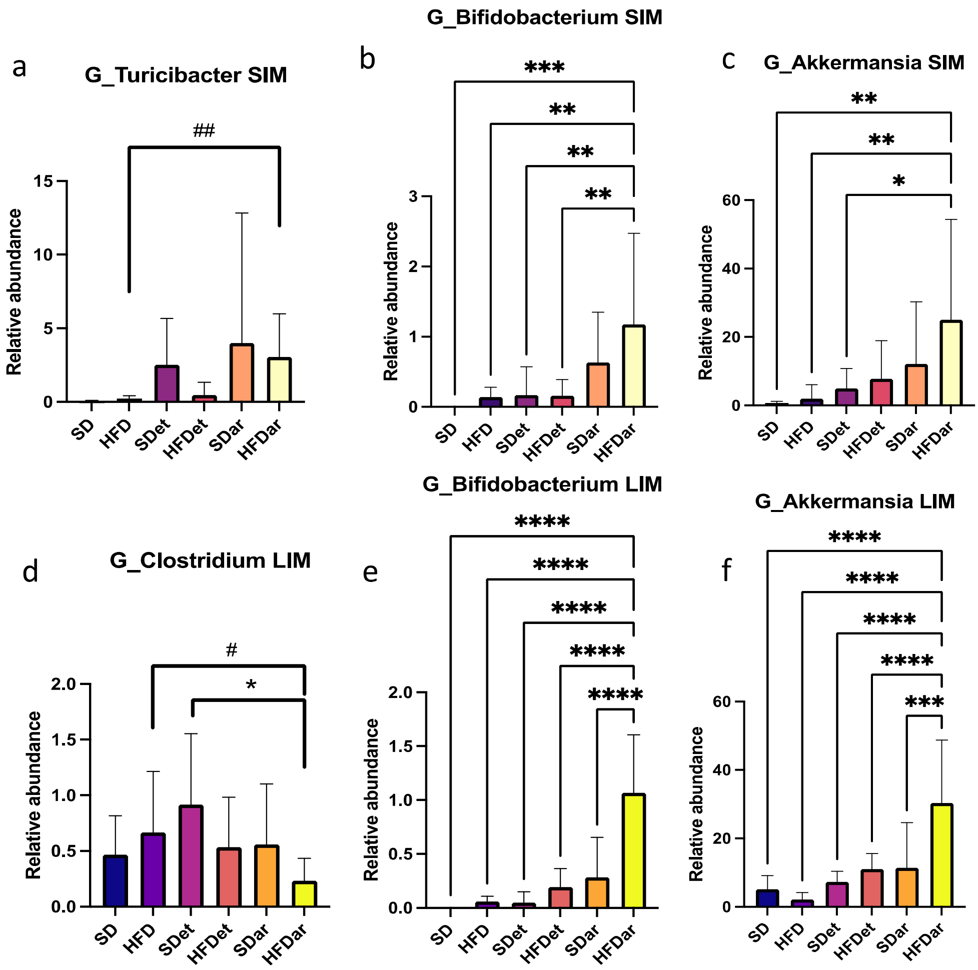

Supplementary Figure S8. Effects of different diet types on mouse SIM and LIM communities at the genus level. (**a**) *Turicibacter* in SIM, (**b**) *Bifidobacterium* in SIM, (**c**) *Akkermansia* in SIM, (**d**) *Clostridium* in LIM, (**e**) *Bifidobacterium* in LIM, and (**f**) *Akkermansia* in LIM. **P* < 0.05, ***P* < 0.01, ****P* < 0.001, *****P* < 0.0001 for ANOVA; # *P*< 0.05, ## *P*< 0.01 for Welch's t test. Comparisons were carried out using one-way analysis of variance followed by Tukey's multiple comparison test or Welch's t test. SD, standard diet; HFD, high-fat diet; SDet, standard diet with solvent addition; HFDet, high-fat diet with solvent addition; SDar, standard diet with C15 supplementation; HFDar, high-fat diet with C15 supplementation.

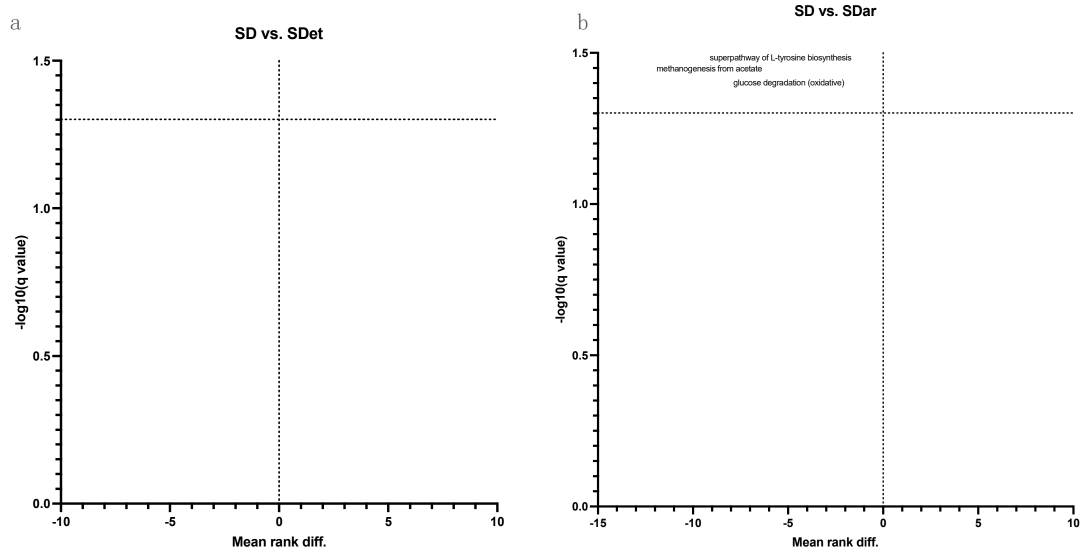

Supplementary Figure S9. Volcano plots demonstrating differences in the representation of metabolic pathways associated with the mouse gut SI microbiota based on multiple Mann–Whitney tests of C57BL/6 mice fed a standard diet (SD) compared to those fed a standard diet (SD) supplemented with C15 (SDar) (**a**) or an SD + solution diet (SDet) (**b**). The volcano plot represents changes in the abundance of metabolic pathways between different dietary conditions. The Q value reflects a false discovery rate of 5%. The mean rank difference values reflect the direction of changes in the abundance of metabolic pathways (values less than zero indicate an increased representation of pathways, while values greater than zero indicate a decreased representation of pathways in the microbiota of mice fed a diet supplemented with C15). Statistically significant values (*P*<0.01) are indicated.

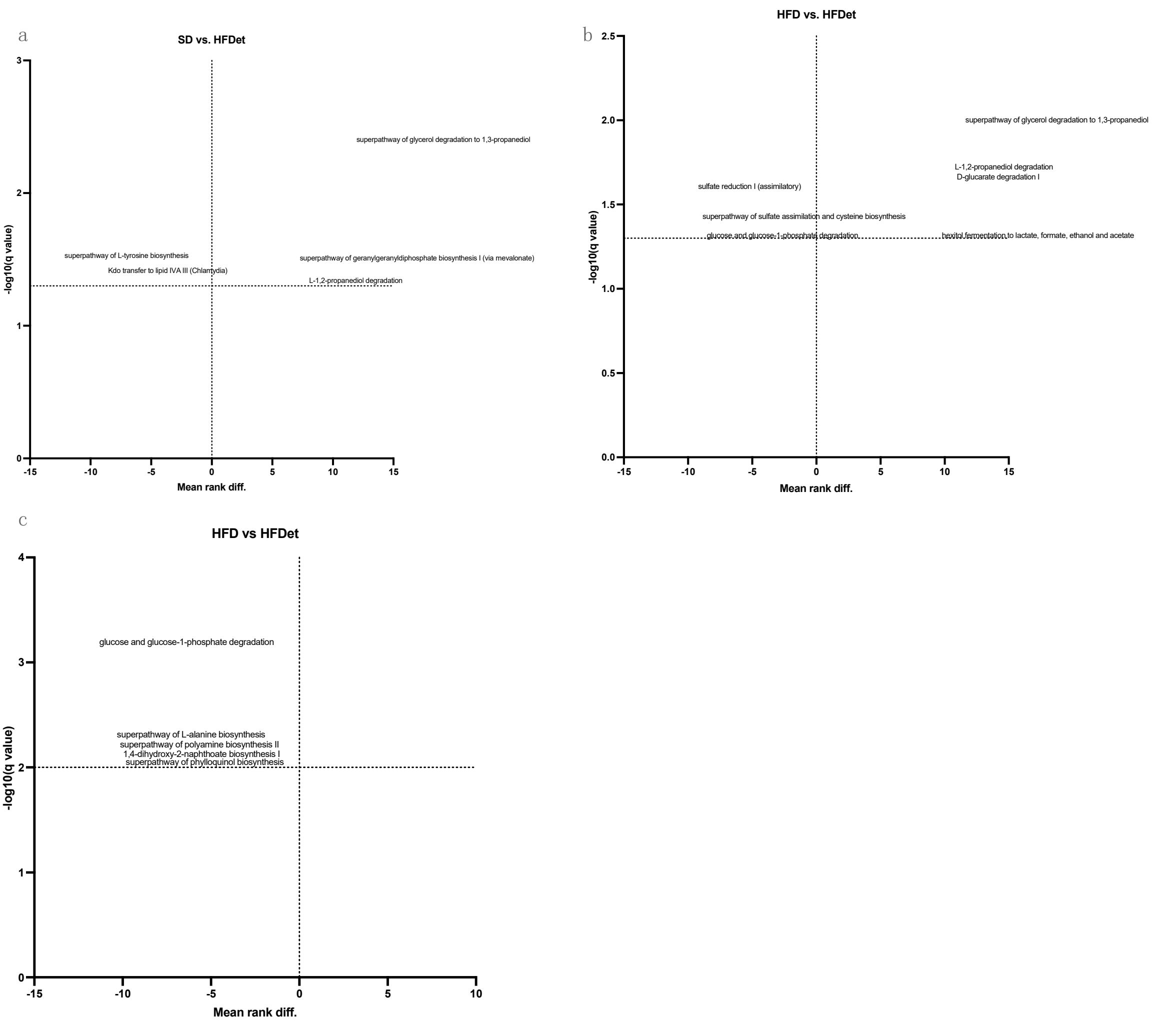

Supplementary Figure S10. Volcano plots demonstrating differences in the representation of metabolic pathways associated with the mouse gut SI and LI microbiota based on multiple Mann–Whitney tests. (a) Differences in representation of SI microbiota metabolic pathways of C57BL/6 mice fed a standard diet (SD) compared to those fed a high fat diet with solvent (HFDet); (b) differences in representation of SI microbiota metabolic pathways of C57BL/6 mice fed a high fat diet (HFD) compared to those fed a high fat diet with solvent (HFDet); (c) differences in representation of LI microbiota metabolic pathways of C57BL/6 mice fed a high fat diet (HFD) compared to those fed a high fat diet with solvent (HFDet). The volcano plot represents changes in the abundance of metabolic pathways between different dietary conditions. The Q value reflects a false discovery rate of 5%. The mean rank difference values reflect the direction of changes in the abundance of metabolic pathways (values less than zero indicate an increased representation of pathways, while values greater than zero indicate a decreased representation of pathways in the microbiota of mice fed a diet with solvent). Statistically significant values (*P*<0.01) are indicated.
