# Supplementary material for: Supplementation of a High-Fat Diet with Pentadecylresorcinol Increases the Representation of Akkermansia muciniphila in the Mouse Small and Large Intestines and May Protect against Complications Caused by Imbalanced Nutrition
Source: Int J Mol Sci. 2024 Jun 15;25(12):6611. doi: 10.3390/ijms25126611 (PMC11204153; doi:10.3390/ijms25126611)
Supplement: Supplementary file 1 [file ijms-25-06611-s001.zip › Supplementary Table S1.pdf]

Supplementary Table S1. Sampling of intestinal contents for metagenomic sequencing analysis

Group of animals	Type of diet	Number of samples obtained for metagenome sequencing analysis	
		Small intestine	Large intestine
1	Regular (standard) chow diet (SD)	12	12
2	High-fat diet (HFD)	12	12
3	Regular (standard) chow diet + ethanol (SDet)	12	12
4	High-fat diet + ethanol (HFDet)	12	12
5	Regular (standard) chow diet + ethanol + C15 (SDar)	11	12
6	High-fat diet + ethanol + C15 (HFDar)	12	12
