# Supplementary material for: Supplementation of a High-Fat Diet with Pentadecylresorcinol Increases the Representation of Akkermansia muciniphila in the Mouse Small and Large Intestines and May Protect against Complications Caused by Imbalanced Nutrition
Source: Int J Mol Sci. 2024 Jun 15;25(12):6611. doi: 10.3390/ijms25126611 (PMC11204153; doi:10.3390/ijms25126611)
Supplement: Supplementary file 1 [file ijms-25-06611-s001.zip › Supplementary table S2.pdf]

Supplementary Table S2. Primers used to amplify the V3-V4 region of bacterial 16S rRNA gene.

Forward Primer:	**TCGTCGGCAGCGTCAGATGTGTATAAGAGACAG**CCTACGGGAGGCAGCAG
Reverse Primer:	**GTCTCGTGGGCTCGGAGATGTGTATAAGAGACAG**GACTACAAGGGTATCTAATCC
