# Supplementary material for: Supplementation of a High-Fat Diet with Pentadecylresorcinol Increases the Representation of Akkermansia muciniphila in the Mouse Small and Large Intestines and May Protect against Complications Caused by Imbalanced Nutrition
Source: Int J Mol Sci. 2024 Jun 15;25(12):6611. doi: 10.3390/ijms25126611 (PMC11204153; doi:10.3390/ijms25126611)
Supplement: Supplementary file 1 [file ijms-25-06611-s001.zip › Supplementary table S3.pdf]

Supplementary Table S3. Beta diversity indexes evaluating differences between small and large intestine microbiota communities in different diet groups of mice. Mean and standard deviation (*SD*) values are shown.
SD, standard diet; HFD, high fat diet; SDet, standard diet + ethanol; HFDet, high fat diet + ethanol; SDar, standard diet + pentadecylresorcinol; HFDar, high fat diet + pentadecylresorcinol.

	**SD**		**HFD**		**SDet**		**HFDet**		**SDar**		**HFDar**	
	Mean	*SD*	*Mean*	*SD*	*Mean*	*SD*	*Mean*	*SD*	*Mean*	*SD*	*Mean*	*SD*
Bray-Curtis Distance	0,700	0,094	0,672	0,104	0,712	0,150	0,698	0,176	0,712	0,120	0,622	0,149
Canberra Distance	1211,626	318,875	1258,741	237,264	1168,039	112,867	1277,055	222,245	1360,248	182,993	1444,696	268,908
Chebyshev Distance	6856,667	9096,835	4239,250	2325,739	6456,750	4822,668	12950,083	13006,116	10027,364	7995,508	3902,917	2380,946
Correlation Distance	0,584	0,207	0,567	0,242	0,739	0,205	0,631	0,283	0,706	0,226	0,475	0,310
Cosine Distance	0,584	0,207	0,567	0,242	0,739	0,205	0,631	0,283	0,706	0,226	0,475	0,310
Euclidean Distance	9512,421	8371,342	7006,025	3381,316	9557,788	6532,752	14586,188	12243,116	13061,397	7902,104	6529,529	2830,590
Jensen-Shannon Metric	0,568	0,092	0,560	0,067	0,602	0,109	0,599	0,132	0,599	0,090	0,532	0,114
Manhattan Distance	41912,750	13491,535	41072,000	12017,555	44533,583	14383,059	45003,583	18841,371	57441,455	22196,615	38627,333	11834,240
Minkowski Distance	9512,421	8371,342	7006,025	3381,316	9557,788	6532,752	14586,188	12243,116	13061,397	7902,104	6529,529	2830,590
Squared Euclidean Distance	154725572,083	333301968,376	59564907,500	67418919,655	130471746,583	195290241,209	350159618,917	477709986,773	227366670,182	266096323,332	49979306,333	43283190,060
